# Task shifting in the care for patients with hand osteoarthritis. Protocol for a randomized controlled non-inferiority trial

**DOI:** 10.1186/s12891-021-04019-9

**Published:** 2021-02-16

**Authors:** Ingvild Kjeken, Kjetil Bergsmark, Ida K. Haugen, Toril Hennig, Merete Hermann-Eriksen, Vivian Tryving Hornburg, Åshild Hove, Anne Prøven, Trine Amalie Sjøvold, Barbara Slatkowsky-Christensen

**Affiliations:** 1 National Advisory Unit on Rehabilitation in Rheumatology, Division of Rheumatology and Research, Diakonhjemmet Hospital, PO Box 23, Vinderen, N-0319 Oslo Norway; 2 Division of Rheumatology and Research, Diakonhjemmet Hospital, PO Box 23, N-0319 Vinderen, Oslo Norway; 3 Martina Hansens Hospital, Sandvika, Norway; 4 Division of Clinical Service, Diakonhjemmet Hospital, Oslo, Norway

**Keywords:** Hand osteoarthritis, Occupational therapy, Exercises, Assistive devices, Orthoses, Cost-effectiveness

## Abstract

**Background:**

Current health policy states that patients with osteoarthritis (OA) should mainly be managed in primary health care. Still, research shows that patients with hand OA have poor access to recommended treatment in primary care, and in Norway, they are increasingly referred to rheumatologist consultations in specialist care. In this randomized controlled non-inferiority trial, we will test if a new model, where patients referred to consultation in specialist health care receive their first consultation by an occupational therapy (OT) specialist, is as safe and effective as the traditional model, where they receive their first consultation by a rheumatologist. More specifically, we will answer the following questions:

1. What are the characteristics of patients with hand OA referred to specialist health care with regards to joint affection, disease activity, symptoms and function?
2. Is OT-led hand OA care as effective and safe as rheumatologist-led care with respect to treatment response, disease activity, symptoms, function and patient satisfaction?
3. Is OT-led hand OA care equal to, or more cost effective than rheumatologist-led care?
4. Which factors, regardless of hand OA care, predict improvement 6 and 12 months after baseline?

**Methods:**

Participants will be patients with hand OA diagnosed by a general practitioner and referred for consultation at one of two Norwegian departments of rheumatology. Those who agree will attend a clinical assessment and report their symptoms and function in validated outcome measures, before they are randomly selected to receive their first consultation by an OT specialist (*n* = 200) or by a rheumatologist (n = 200). OTs may refer patients to a rheumatologist consultation and vice versa. The primary outcome will be the number of patients classified as OMERACT/OARSI-responders after six months. Secondary outcomes are pain, function and satisfaction with care over the twelve-month trial period. The analysis of the primary outcome will be done by logistic regression. A two-sided 95% confidence interval for the difference in response probability will be formed, and non-inferiority of OT-led care will be claimed if the upper endpoint of this interval does not exceed 15%.

**Discussion:**

The findings will improve access to evidence-based management of people with hand OA.

**Trial registration:**

ClinicalTrials.gov, NCT03102788. Registered April 6th, 2017, https://clinicaltrials.gov/ct2/show/NCT03102788?term=Kjeken&draw=2&rank=1

**Date and version identifier:**

December 17th, 2020. First version.

## Background

The Global Burden of Disease project reports osteoarthritis (OA) as one of the largest causes of years lived with disability worldwide [1]. The hand is the most commonly affected joint site [2], with the distal and proximal interphalangeal finger joints and the carpometacarpal (CMC1) joint of the thumb as the most frequently involved joints [3]. Typical symptoms of hand OA are pain on usage and morning or inactivity stiffness, while clinical hallmarks are Heberden and Bouchard nodes and/or bony enlargement with or without deformity. With these typical features, a confident accurate diagnosis can usually be made based on patient history and clinical examination [4].

Prevalence estimates for hand OA varies. In a recent population-based cohort study, the lifetime risk of symptomatic hand OA was 47.2% in women and 24.6% in men [5]. Today, OA is understood as a disease with chronic abnormal remodelling affecting the entire synovial joint organ. The outcomes are structural and functional failure, negatively influencing body functions and structures, activity performance, work ability and health related quality of life [6, 7]. Functional impairments of hand OA often equal to those of rheumatoid arthritis, but currently there are fewer treatment options established and available [8]. There is also a well-known discordance between symptoms, functional impact and radiographic changes, with some people not experiencing symptoms, but showing radiographic changes and vice versa [1].

**Fig. 1:**
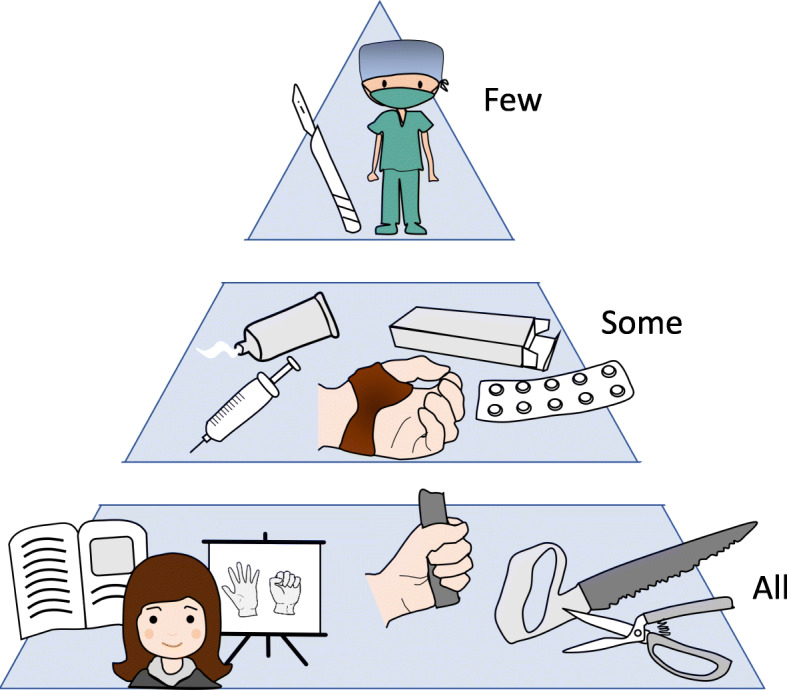
Treatment pyramid for people with hand osteoarthritis. Illustration by Anne Therese Tveter ©

There is yet no cure or disease modifying drugs for OA. Based on updated evidence, the European League Against Rheumatism (EULAR) recommendations state that patient education, hand exercises, assistive devices and orthoses, frequently delivered by occupational therapists (OTs), are the core interventional treatments [9] (Fig. 1).

Topical Non Steroid Anti Inflammatory Drugs (NSAIDs) is the first pharmacological treatment of choice, whereas oral analgesics, particularly NSAIDs, should be considered for a limited duration for symptom relief. Intra-articular injections of glucocorticoids should only be considered in patients with painful interphalangeal joints, and surgery first when other treatment modalities have failed.

In the new health sector reform, the Norwegian Directorate of Health has placed the main responsibility for OA treatment in primary health care [10]. However, there is a significant evidence-to practice gap in OA care. Recent research shows that the quality of primary care services in general is sub-optimal for this patient group [11], and in particular, people with hand OA have poor access to recommended treatment both in primary and secondary care [12]. This is supported by contemporary results from a Norwegian trial, where only 21% of patients had received recommended non-pharmacological treatment before being referred from their general practitioner (GP) to surgical consultation due to CMC1 OA [13]. Thus, patients with hand OA are currently not receiving the recommended intervention options and are increasingly referred to consultation by a rheumatologist in specialist care. At the same time there are often long waiting lists for new rheumatologist appointments, and their time should primarily be spent on patients with inflammatory rheumatic diseases, for whom early diagnosis, medical treatment and tight controls may induce disease remission [14]. New models for hand OA care are therefore needed, in which health professionals in extended roles may fulfil the important role as gatekeepers to facilitate rapid access to appropriate care and shorten wait times to rheumatologists [15].

### Task shifting and extended roles in rheumatology care

The World Health Organization defines task shifting as “a process of delegation whereby tasks are moved, where appropriate, to less specialized health workers”, and argues that reorganizing the workforce is necessary to make more efficient use of the human resources currently available [16]. Tasks may thus be shifted to professions with shorter education who takes on extended roles, or between professions with lower level of specialist knowledge, i.e. between specialist and primary care.

Task shifting in rheumatology care have often been addressed by models that rely on health care providers other than physicians or rheumatologists in extended clinical roles, such as nurse-led care. In a review focusing on rheumatoid arthritis, the authors conclude that nurse-led care is highly acceptable to patients, equally effective, and safe in the short term. The results also indicated that nurse-led care is equal or less costly than other models, but the evidence is insufficient to draw conclusions regarding its accessibility and appropriateness [17].

In two reviews of physiotherapists (PTs) working in extended roles in musculoskeletal care, the authors conclude that even if patients were satisfied with PT-led care, there is a lack of studies assessing the benefits of such roles in terms of costs and clinical outcomes [18, 19].

Concerning the impact of extended roles for allied health professionals, two reviews of the qualitative and quantitative evidence show that new tasks included triaging, referral to other services and prescribing conservative treatment [20, 21]. Therapists found the job stressful but satisfying, and did also identify additional training needs required to effectively carry out these roles. Therapists and physicians further reported concerns in terms of litigation, lack of confidence, variations in training, and the reservation that the extended role service is “only as good as the therapist employed”. The authors conclude that despite the introduction of extended roles for allied health professionals, evidence about the impact of such roles is limited, and that service modernization requires role development and robust evaluation of the outcomes for patients.

Hand OA often affects performance of everyday activities. OTs are therefore in a crucial position to provide tailored care for this patient group, because they are specialized in rehabilitation methods to reduce activity limitations and participation restrictions caused by hand OA, and in client-centered approaches that take into consideration both psychological, social, and environmental needs [22]. There is clear evidence that OTs treating patients with hand OA provide effective interventions benefiting both patients and health services. O’Brien et al. concluded that receiving non-operative treatment by an OT was the only significant predictor for not requiring surgery among 224 patients referred to a hand surgeon for common hand conditions, such as hand OA [23]. In a recent randomized controlled trial (RCT), occupational therapy in the waiting period to surgical consultation led to improved function after three months [24] and less surgery after two years in patients with CMC1 OA [25]. Furthermore, in an ethnographic study exploring the practice in OT-led hand clinics, the authors conclude that the therapists’ occupational perspective on assessing and addressing activity and participation is as an important and added bonus [26].

In Norway, OT-led hand OA care was piloted at St. Olav’s Hospital, Trondheim in 2015–16 [27]. The results showed that 7 of the 24 participants, who ordinarily would have been seen by a rheumatologist, needed a short rheumatologist consultation following their OT consultation, thereby saving 21.5 rheumatologist hours. Seventy-five percent of the participants reported that they were satisfied or very satisfied with OT-led care. Whilst this study led to OT-led hand OA care being implemented as routine care at St. Olav’s Hospital in 2016, it did not evaluate safety or patient outcomes.

## Methods/design

### Aims and research questions

In this randomized controlled non-inferiority trial, we will test if a new model, where patients referred to consultation in specialist health care receive their first consultation by an OT specialist, is as safe, effective and cost-effective as the traditional model, where they receive their first consultation by a rheumatologist.

More specifically, our study will consider the following research questions:

1. What are the characteristics of patients with hand OA referred to specialist health care with regards to joint affection, disease activity, symptoms and function?
2. Is OT-led hand OA care as effective and safe as rheumatologist-led care with respect to treatment response, disease activity, symptoms, function and patient satisfaction?
3. Is OT-led hand OA care equal to, or more cost effective than rheumatologist-led care?
4. Which factors, regardless of hand OA care, predict improvement 6 and 12 months after baseline?

### Trial development

The trial has been designed in line with the SPIRIT guidelines [28], in a project group consisting of the following key stakeholders: Rheumatologists and OTs with experience of treating patients with hand OA, researchers with expertise in hand OA, and two patient research partners (PRPs) with experience from living with hand OA, including receiving pharmacological, surgical and non-pharmacological interventions. The PRPs also gave input to ensure an optimal recruitment process. Members of the project group will be engaged throughout each stage of the trial and will contribute in the process of integrating study results in clinical practice.

### Study design and setting

This is a Norwegian multicentre randomized controlled non-inferiority trial. This design is suitable to show that the new treatment is not an unacceptably worse alternative to the standard. The trial will be led by the National advisory unit on rehabilitation in rheumatology, which is located at Diakonhjemmet Hospital in Oslo, and participants will be included at the departments of rheumatology at Martina Hansen’s Hospital and Diakonhjemmet Hospital.

### Participants

Patients with hand OA diagnosed by a GP and referred for consultation at Martina Hansen’s Hospital or Diakonhjemmet Hospital are eligible for the RCT and will from September 1st 2017 be invited to participate. Inclusion criteria are age ≥ 18 years, and having a good understanding of Norwegian. Exclusion criteria are psoriasis, cognitive impairment or severe psychiatric disorder, or possible inflammatory rheumatic disease (erythrocyte sedimentation rate > 40 mm/hour or C-reactive protein> 20 mg/L). Patients’ informed consent will be sought and given the local project coordinator prior to data collection.

The local project coordinator at each hospital will have access to lists of patients who are referred for consultation and will mail written information about the study to potential participants. Within the next week, the coordinator will call each person to give additional information, answer questions, screen for inflammatory rheumatic disease and, if eligible, invite her/him to participate in the study. Those who agree will be booked for an appointment for the baseline assessment, followed by a consultation with either a rheumatologist or an OT the same day. A questionnaire and a consent form will be sent to the participant, who will be encouraged to fill it out and bring it to the appointment. The coordinator will also make sure that blood samples and hand x-rays are collected prior to baseline assessment and that the results are uploaded into the patient’s medical record before she/he arrives at the hospital.

### Data collection

**Fig. 2:**
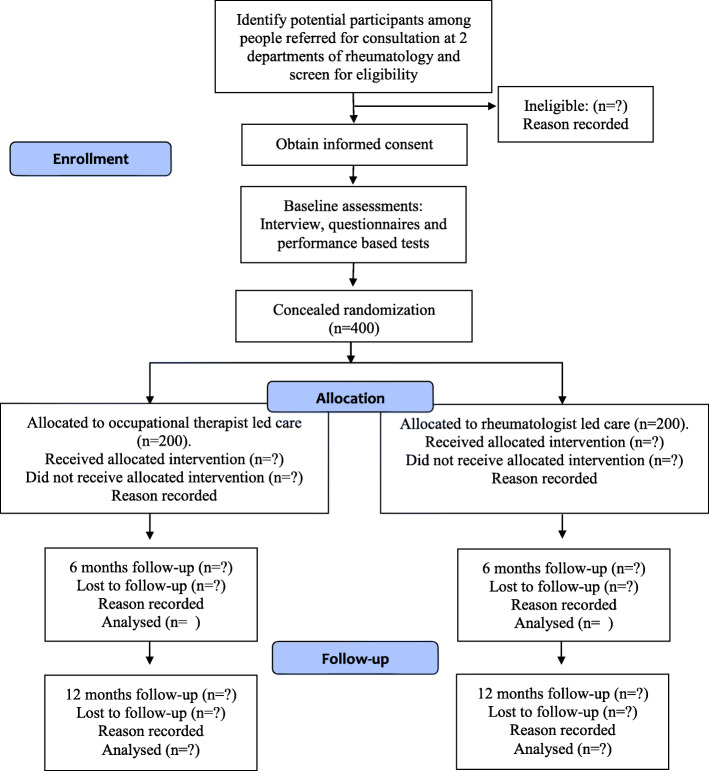
CONSORT flow diagram

Participant flow is shown in Fig. 2. On arrival at the centre, a research assistant (an OT or PT) not involved in the recruitment or treatment of participants will perform the baseline assessment before the participant is randomised to one of the two treatment arms and thereafter, dependent on allocation, consults with a rheumatologist or an OT.

Follow-up will be at 6 and 12 months to evaluate short and long-term effects. Before the 6 month follow-up, a questionnaire will be sent to the participants, who will be asked to complete it at home and bring it to the follow-up appointment. To achieve observer blinding, patients will be asked not to inform the assessor about their group allocation in the 6-month assessments. Follow-up at 12 months is based on patient reported outcomes only. Each participant will receive a questionnaire and a prepaid envelope, and is asked to fill in the questionnaire and return it to the hospital in the envelope.

Each participant will have a special numeric code that will be used to link the data from follow-ups to baseline data, and all data will be stored in a fireproof and locked cupboard and in a digital file on the research server at Diakonhjemmet Hospital. Selected members of the project group (project leader, local coordinators, bio-statistician, study assistants, and a postdoctoral research fellow) can access the file. The OT or PT performing the baseline and follow-up assessment at 6 months will check and ensure that there are no missing data in the questionnaires. An independent study assistant will check incorrect and double data entries.

### Randomization, allocation concealment and blinding

A statistician not involved in the study will make two computer-generated randomisation list with a block size of 10, one for each centre. A secretary will prepare sealed, opaque envelopes containing the patient’s assignment to either rheumatologist-led or OT-led care. The envelopes will be stored in a locked closet at each centre and will be opened by the research assistant after baseline assessments are completed. In this trial, patients and clinicians delivering the intervention will be aware of the treatment assigned. However, the OTs and PTs performing baseline and follow-up assessments and the statistician performing the main statistical analyses will be blinded to group allocation.

### Ethical considerations

The study will take place in specialist care to allow for detailed monitoring of the safety of OT-led care. This will be done in a three step process. First, all GP referrals will be reviewed by a rheumatologist, who will remove patients with a possible inflammatory rheumatic disease from the list of eligible participants. Secondly, the local project coordinator will screen potential participants for psoriatic arthritis in a telephone call prior to inclusion. Third, the consulting OT will evaluate radiographs and blood samples and perform a clinical examination. If this leads to doubt regarding the hand OA diagnosis, the patient will be examined by a rheumatologist after the OT-consultation to exclude a diagnosis of inflammatory arthritis. Regarding research ethics, the study will be conducted according to the Declaration of Helsinki. Personal confidentiality will be guaranteed, and declarations of voluntary participation with detailed information on the data collection will be signed by each participant, emphasising the right to withdraw from the project at any time without any explanation. The study has been reviewed by the Norwegian Regional Committee for Medical Research Ethics (2017/742/REK sør-øst A), and is registered in the Clinical Trials register (NCT03102788). Further, the data protection officer at Diakonhjemmet Hospital have ensured that the study comply with the requirements in the General Data Protection Regulation, (2020/00184). Participants who suffer harm from trial participation will be provided compensation trough The Norwegian System of Patient Injury Compensation .

### Confidentiality

All collected data will be regarded as confidential and will be securely stored in paper formats in a locked closet in a locked room. The project leader shall regularly remain in touch with the local project coordinators and provide assistance in the data collection process, in addition to ensuring that the data are collected, stored and quality assured in accordance with current guidelines from the Norwegian Regional Committee for Medical Research Ethics. The project leader, a research assistant, a bio-statistician, two PhD students and a post doc candidates will have access to the final trial dataset.

### Intervention providers

Four certified OT specialists (two at each centre) will provide the OT-led care, whereas rheumatologists at each centre will provide the rheumatologist-led care.

Before the start of the inclusion process, the research assistants will meet together with the project leader and the patient research partners and go through assessment procedures to ensure that these are performed consistently. Further, the OT specialists will meet and agree on the main elements in OT-led care. The rheumatologists will continue to deliver usual care. Meetings with the project group will be held regularly throughout the trial period. Additionally, the project leader will answer questions from those involved in the study by mail or telephone between meetings.

### Treatment interventions

Participants in rheumatologist-led care will receive their first consultation by a rheumatologist, whereas participants in OT-led care will receive their first consultation by an OT specialist. Rheumatologists may, however, refer patients to an OT consultation (or other services) and vice versa, and both groups will receive treatment as usual.

### Rheumatologist-led care

Rheumatologist-led care will follow international recommendations for hand OA treatment [29–31]. All participants will receive oral information about hand OA and undergo a clinical examination. Depending on the patient’s symptoms, comorbidities, and localisation and severity of hand OA, she/he will receive information about pharmacological treatment, intra-articular injections of glucocorticoids, and/or referral to OT, surgical consultation or other relevant procedures or professions. The consultation will last 45 min.

### Occupational therapist-led care

The OT-intervention will be based on international recommendations for hand OA treatment [29–31], and studies on hand OA care [12, 32–35]. All participants will undergo a clinical examination and receive oral and written information (in a booklet) about hand OA and use of medication; ergonomic principles in activities of daily living and use of assistive devices; use of orthoses and instruction in hand exercises. Additionally, those with CMC1 OA will receive orthoses if needed and be referred to surgical consultation if relevant, whereas participants with clear inter-phalangeal or CMC1 inflammation may be referred to a rheumatologist for intra-articular glucocorticoid injections. The content of the OT-intervention as set up in the booklet is detailed in Table 1. The OT-consultation will last 45 min, but patients may return for a second consultation if needed.

**Table 1:**
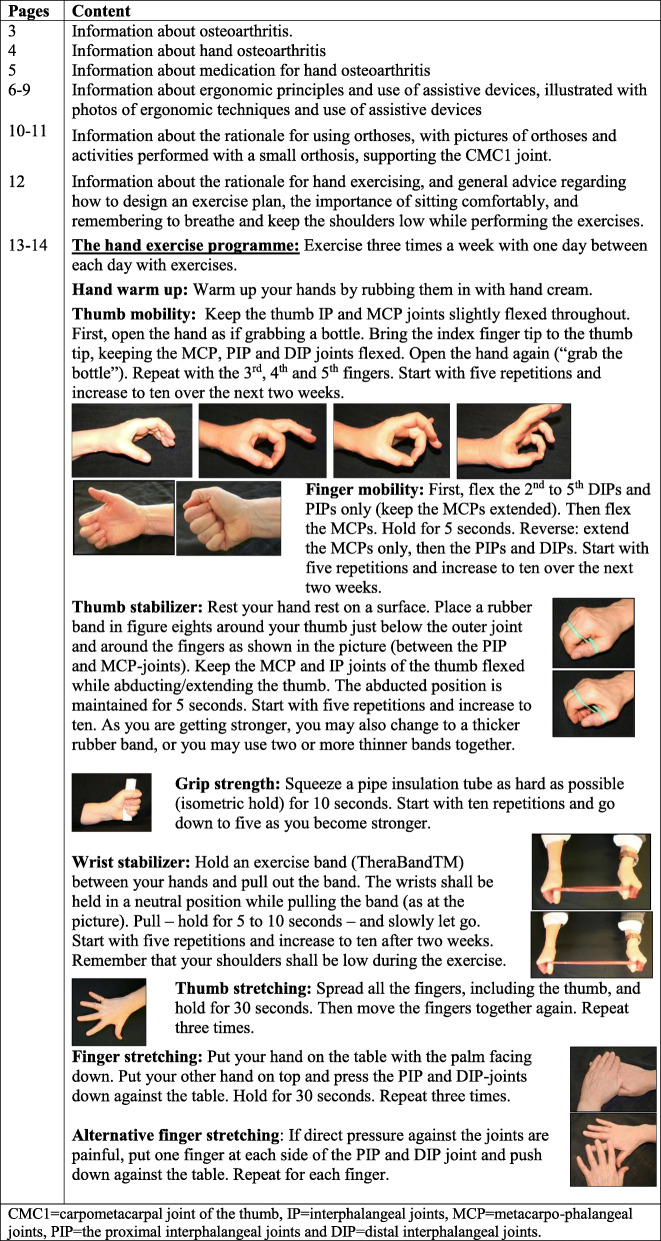
Occupational therapy intervention, as described in the booklet for patients in occupational therapist led care

### Other interventions

Participants at Diakonhjemmet Hospital will be invited to participate in the hospitals’ one-day multidisciplinary group-based educational program for people with hand, hip or knee OA, and in a hand exercise group. The group is led by an OT, who supervises up to ten participants in performing the exercises in the program in the booklet (Table 1). The session will last approximately 60 min, and participants may come back for an additional group session after two weeks.

### Descriptive and outcome measures

To be able to describe and compare patient groups, information on socio-demographics (age, gender, marital status, education, occupational status,), height and body weight, number of interphalangeal joints with bony enlargement (0–14 in each hand), degree of hand OA (on basis of conventional radiography of both hands graded according to the Kellgren-Lawrence method [36]), co-morbidity and medication will be collected at inclusion.

Outcome measures will be in line with the internationally recommended OMERACT core set for OA [37]. The primary outcome will be OMERACT/OARSI response at six months. This is a composite index summarizing change in pain, function and patient’s global assessment. The responder classification is reported as a single variable (responder yes/no) [38]. Secondary outcomes are documented in Table 2 and will be based on previously validated measures.

**Table 2:** Summary of measures to be collected

	Data collection instrument and scale	Time points
**Primary outcome measure:**		
Number of patients classified as OMERACT/OARSI-responders in each group.	The responder classification is a composite index reported as a single variable (responder yes/no), based on change after treatment in pain, function and patient’s global assessment.	t2, t3
**Secondary outcomes:**		
Hand pain at rest	Numeric rating scale: 0–10, 0 is no pain	t1, t2, t3
Physical function	The Functional Index for Hand OsteoArthritis is a summary score (ranging from 0 to 30) of 10 items, each scored on a four-point scale, 0 = possible without difficulty	t1, t2, t3
Patient’s global assessment	Numeric rating scale: 0–10, 0 is no disease activity	t1, t2, t3
Activity performance	In the Measure of Activity Performance of the Hand, as a mean of 18 standardised activities, (rating scale 1–4, 1 is no problems)	t1, t2, t3
Maximal grip strength	In kg by the JAMAR dynamometer	t1, t2,
Painful finger joints	Examination by a trained OT of presence of pain yes/no in CMC1, MCP, PIP and DIP-joints in both hands	t1, t2
Pain in activity at each hand (measured following measurement of grip strength)	Numeric rating scale: 0–10, 0 is no pain	t1, t2,
Hand stiffness	Numeric rating scale: 0–10, 0 is no stiffness	t1, t2, t3
Health related quality of life	EuroQoL 5D-5 L - health-related quality of life contains five items scored at a five level scale, (“no problems” to “unable to do”) and a visual analogue scale ranging from 0 and 100. 0 corresponds to “the worst health you can imagine”.	
Satisfaction with care	The PasOpp questionnaire contains eleven statements with a 5-point scoring scale ranging from “not at all” to “very much”, and one statement concerning waiting time with a 4-point scoring scale ranging from “not at all” to “very long”.	t2,

t1 = baseline, t2 = 6 months after baseline, t3 = 12 months after baseline. OT = occupational therapist. CMC1 = carpometacarpal joint of the thumb, MCP = metacarpal joint, PIP = proximal interphalangeal, DIP = distal interphalangeal, IP = interphalangeal joint of the thumb

Patient-reported outcome measures will be collected at baseline, 6 and 12 months and will include the following symptoms, functional aspects and measures: Pain at rest and in activity, hand stiffness and patient’s global assessment of disease activity on 0–10 numeric ratings scales; activity performance measured by the Measure of Activity Performance of the Hand (MAP-Hand) [24, 39]; physical function measured by the Functional Index for Hand OsteoArthritis (FIHOA) [40]; and health related quality of life with the EQ-5D-5L [41]. Satisfaction with care will be recorded by the Patient Experiences (PasOpp) questionnaire [42].

Additionally, the following observer-reported outcome measures will be reported at baseline and 6 months: number of painful joints (0–9 in each hand), and grip strength measured with the JAMAR dynamometer [43].Information about adverse events will be collected from patients’ medical records and used in the evaluation of safety.

To allow for analyses of cost-effectiveness, participants will at six and 12 months report costs related to their hand OA over the period since previous assessment/control: medication; number of days of sick leave from paid work; number of visits to a given list of health providers, and number of hospital visits or stays. Additionally, data on health-care resource allocation and costs related to received treatment will be collected at each hospital. An important way of assessing the effects of treatment in health economic evaluations is the use of utility indexes. In this study we will use the EQ-5D-5L as a utility measure in the cost-effectiveness analyses.

### Sample size calculations

The primary outcome will be number of patients classified as OMERACT/OARSI-responders after six months [38]. We have not been able to identify any study examining the effect of rheumatologist-led care for hand OA, but in a double-blind, randomized, placebo-controlled study with 70 participants, the effect of low-dose oral prednisolone in hand OA was assessed using the responder criteria [44]. A total of 20 participants (57%) in each group met the responder criteria after four weeks. However, it is likely that the percentage of responders declines over time. Based on discussions in the project group, we have conservatively estimated that the percentage of responders after six months in rheumatologist-led care will be 35%. With a non-inferiority margin set to 15%, 80% statistical power, a two-sided 95% confidence interval, and an estimated drop-out rate of 20%, we estimate that 400 patients (200 in each arm) will provide sufficient power to exclude that OT-led care is inferior to rheumatologist led care, assuming a response probability of 35% in each group.

### Statistical analyses

The primary analyses will be conducted blinded to treatment allocation and will be carried out according to the intention-to-treat principle. The analysis of the primary outcome, OMERACT/OARSI response at six months, will be done by logistic regression. A two-sided 95% confidence interval (CI) for the difference in response probability (rheumatologist-led care minus OT-led care) will be formed, and the non-inferiority of OT-led care will be claimed if the upper endpoint of this interval does not exceed 15%. Per-protocol analyses will also be performed.

In terms of secondary outcomes, differences in mean values with 95% CI at each follow up will be analysed using the linear mixed model, adjusting for baseline levels of the outcome measure.

To assess the cost-effectiveness, we will need to estimate health outcomes and costs. The health outcomes will be measured using the EQ-5D-5L and the costs will include the cost of the two interventions. This comprises the rheumatologist and OT hours, costs related to intra-articular injections and provision of orthoses, and costs related to other treatment (i.e. patient education or hand exercise group). Furthermore, costs related to medical or technical equipment purchased by participants and to the use of other health care services (primary care services, rehabilitation, and institution) will be recorded for both groups during the trial period. Standard methods for economic evaluation will be applied and the cost-effectiveness will be calculated as the incremental cost-effectiveness ratio, which is defined by the cost per incremental QALY. All analyses will be performed using Stata 16.0.

### Dissemination of study results

We plan to publish at least four articles in international peer-reviewed journals using the data collected in this study. These will be written by a postdoctoral research fellow, and correspond to the research questions of the study. Authorship will be based on scientific contribution and enrollment, according to the guidelines set forth in the Vancouver protocol.

The results will also be disseminated through the information channels of the project group members, including web sites, national and international multidisciplinary networks of health professionals, general practitioners and rheumatologists, and national and international conferences and congresses.

Lecturing at bachelor, master and PhD programs will be done to educate young clinicians and researchers. To reach patients and their relatives, the results will be published in the journal of the Norwegian Rheumatism Association, and at conferences and meetings arranged by the association.

We also plan to arrange a national conference for rheumatologists, health professionals, members of the Norwegian Rheumatism Association, leaders of bachelor, master and PhD programs for health professionals, and representatives from the national associations for nurses, OTs, PTS and GPs where the results will be presented and discussed, together with plans for an implementation study.

## Discussion

Hand OA is one of the most common joint conditions, and occupational therapists play an important role in the treatment of this patient group [22]. As there is no cure for hand OA, there is a need to develop effective treatment approaches that achieve sustainable long-term improved outcomes. This paper describes the rationale and design of a trial investigating if OT-led care is as safe, effective and cost-effective as traditional rheumatologist-led care with respect to treatment response and patient satisfaction. The design of the trial is based on international evidence-based recommendations for hand OA, and the aim is to improve access to safe and effective care, professional practice and cost-effective utilization of health care resources.

The study has been developed in close collaboration with patient research partners, clinicians and researchers, who will also contribute in the process of integrating study results in clinical practice. To our knowledge, it is one of very few large randomized controlled trial exploring the safety and feasibility of a new model of care for people with hand OA. The results will also provide insight into referral practice, as well as predictors for improving subsequent therapy. If deemed safe and cost-effective, the model may be implemented in a larger scale in secondary care, thereby contributing to reduced variability and increased health equity for people with hand OA. Such a model may also reduce waiting time for rheumatologist consultation for people with inflammatory rheumatic diseases, for whom early diagnosis, disease modifying medication and tight controls may induce remission and prevent irreversible joint damage and long-term disability.

In Norway, as in many other countries, task shifting has been seen as a prerequisite for successful establishment of new primary health care models [45], to ensure future quality of specialist health care services [46], and to implement evidence-based and effective interventions for large patient populations [47]. In a paper from 2009, Dziedzic and colleagues call for new models of OA care and suggest that primary care is the ideal arena to achieve high-impact secondary prevention of pain and disability in this patient group [22]. Interventions such as patient education, assistive devices and hand exercises may easily be provided in primary care, thereby reducing the need for treatment in secondary care. Further, a closer collaboration between GPs and primary care therapists may result in patients receiving recommended treatment earlier in the hand OA disease trajectory, when there is a larger potential for preventing or reducing functional limitations.

The results concerning safety, effect and cost-effectiveness may also be relevant for task shifting in the care of other chronic diseases, thereby enhancing professional practice and cost-effective utilisation of health care resources in the treatment of large patient groups. The inclusion of an economic evaluation in our trial offers an additional dimension that will assist health policymakers in their decision-making regarding which models of care that is most feasible for people with hand OA.

## Data Availability

Data will be available from the corresponding author upon reasonable request.

## Abbreviations

CI: Confidence interval
CMC: Carpometacarpal
DIP: Distal interphalangeal
EQ-5D-5L: EuroQoL 5 L - health-related quality of life
EULAR: the European League Against Rheumatism
FIHOA: Functional Index for Hand OsteoArthritis
GP: General practitioner
IP: Interphalangeal
MAP-Hand: Measure of Activity Performance of the Hand
MCP: Metacarpophalangeal
NSAIDs: Topical Non Steroid Anti Inflammatory Drugs
OA: Osteoarthritis
OARSI: Osteoarthritis Research Society International
OMERACT: Outcome Measures in Rheumatology
OT: Occupational therapy
PIP: Proximal interphalangeal
PRP: Patient research partner
PT: Physiotherapist
QALY: Quality-adjusted life years
RCT: Randomized controlled trial
